# Incidence and predictors of problem gambling in first-episode psychosis: A prospective multicentre cohort study

**DOI:** 10.1192/j.eurpsy.2026.10167

**Published:** 2026-02-13

**Authors:** Olivier Corbeil, Maxime Huot-Lavoie, Amal Abdel-Baki, Laurent Béchard, Sébastien Brodeur, Laurence Artaud, Prométhéas Constantinides, Christian Jacques, Jean-François Morin, Clairélaine Ouellet-Plamondon, Marco Solmi, Denis Talbot, Michel Dorval, Isabelle Giroux, Marc-André Roy, Marie-France Demers

**Affiliations:** 1 Faculty of Pharmacy, Université Laval, Quebec, Canada; 2 Institut universitaire en santé mentale de Québec (IUSMQ), Quebec, Canada; 3 CERVO Brain Research Centre, Quebec, Canada; 4 Department of Psychiatry, Faculty of Medicine, Université Laval, Quebec, Canada; 5 Département de psychiatrie et d’addictologie, Faculty of Medicine, Université de Montréal, Quebec, Canada; 6 Centre de recherche du Centre hospitalier de l’Université de Montréal (CRCHUM), Quebec, Canada; 7 Youth mental health service, Department of Psychiatry, Centre hospitalier de l’université de Montréal (CHUM), Quebec, Canada; 8 Faculty of Nursing, Université Laval, Quebec, Canada; 9 École de psychologie, Université Laval, Quebec, Canada; 10 Centre québécois d’excellence pour la prévention et le traitement du jeu, Quebec, Canada; 11 SCIENCES lab, Department of Psychiatry, University of Ottawa, Ontario, Canada; 12 Regional Centre for the Treatment of Eating Disorders and On Track: The Champlain First Episode Psychosis Program, Department of Mental Health, The Ottawa Hospital, Ontario, Canada; 13 Ottawa Hospital Research Institute (OHRI), Clinical Epidemiology Program, University of Ottawa, Ontario, Canada; 14 Department of Child and Adolescent Psychiatry, Charité Universitätsmedizin, Berlin, Germany; 15 Department of Social and Preventive Medicine, Faculty of Medicine, Université Laval, Quebec, Canada; 16 CHU de Québec – Université Laval Research Centre, Population Health and Optimal Health Practices Program, Quebec, Canada; 17 CHU de Québec – Université Laval Research Centre, Oncology Program, Quebec, Canada

**Keywords:** aripiprazole, early intervention, gambling, incidence, psychosis, risk factors, schizophrenia

## Abstract

**Background:**

Psychiatric comorbidities are common in first-episode psychosis (FEP) and hinder recovery. Problem gambling (PBG), despite potentially serious clinical consequences, remains under-investigated in this population. This study aimed to estimate the incidence of PBG in FEP and identify predictive factors.

**Methods:**

This prospective cohort study was conducted at two FEP programmes in Quebec, Canada. Individuals aged 18–35 years diagnosed with FEP between November-1-2019 and January-31-2023 were screened for PBG using the Problem Gambling Severity Index through May-1-2023. The primary outcome was incident PBG. Time-varying Cox regression models were used to estimate hazard ratios (HRs) for candidate predictors.

**Results:**

Among 520 individuals without prior PBG (mean age = 24.6±4.0 years; 28.8% women), 18 developed PBG during a mean follow-up of 478 days, yielding an incidence rate of 2.6 cases/ 100 person-years. In site-adjusted analyses, white ethnicity (HR = 9.7; 95%CI = 1.3–74.8), incomplete high school education (HR = 2.8; 95%CI = 1.1–7.2), stimulant use disorder (HR=2.8; 95%CI = 1.0–7.3), use of D2/D3-5-HT1A partial agonists (HR = 4.6; 95%CI = 1.5–14.1), and prior non-problematic gambling (HR = 3.1; 95%CI = 1.1–8.4) predicted increased risk. Thirteen cases occurred during aripiprazole treatment, which remained associated with increased PBG risk after multivariable adjustment (adjusted HR = 4.7; 95%CI = 1.6–13.9).

**Conclusions:**

Despite the limited number of incident cases, these results suggest that PBG is relatively common PBG and is associated with potential risk factors, including white ethnicity, incomplete high school education, stimulant use disorder, prior non-problematic gambling, and treatment with D2/D3-5-HT1A partial agonists, particularly aripiprazole. These findings underscore the importance of routine screening for PBG and risk-informed antipsychotic prescribing in FEP.

## Introduction

Psychotic disorders affect ~3% of the population and are associated with substantial disability and a 10–20-year reduction in life expectancy [1–3]. Although antipsychotic medications reduce psychiatric hospitalisations and all-cause mortality [4–6], long-term recovery rates remain low, with fewer than one-third of individuals with first-episode schizophrenia achieving full recovery [7, 8]. Early intervention services (EISs) for first-episode psychosis (FEP) have demonstrated superiority over standard care on several short- and medium-term outcomes, including symptom reduction, functional improvement, return to school or work, and decreased suicide risk [9, 10]. While now considered the standard of care, EISs have nevertheless shown limited effectiveness in improving long-term recovery [8]. As a result, there is a growing consensus that treatment must move beyond symptom control to address both psychiatric and physical comorbidities within an integrated, recovery-oriented framework [11].

Psychiatric comorbidities are highly prevalent in psychotic disorders and represent major barriers to recovery. Among these, behavioural addictions, including problem gambling (PBG), have received limited attention despite emerging evidence of a complex, potentially bidirectional relationship with psychosis [12]. Recent meta-analyses estimate that up to 8.7% of individuals with psychotic disorders experience PBG [13], compared to 1.3% in the general population [14], with reciprocal findings suggesting an elevated prevalence of psychotic disorders among problem gamblers [15]. In the general population, PBG has been associated with male gender/sex, younger age (18–35 years), low educational attainment (i.e., incomplete high school), socioeconomic disadvantage, impulsivity, and substance use disorders (SUDs) [16, 17]. However, it is unclear whether these risk factors operate similarly in individuals with psychotic disorders, particularly during the early stages of illness when neurodevelopmental vulnerabilities and psychosocial stressors may be especially pronounced.

Individuals with FEP may be particularly vulnerable to PBG due to the clustering of these risk factors and the high burden of psychiatric comorbidities in this population. Growing concern also surrounds the potential contribution of third-generation antipsychotics, specifically dopamine D2/D3–5-HT1A partial agonists such as aripiprazole, to the emergence of impulse control disorders and PBG [18–21]. While retrospective studies and pharmacovigilance data have suggested an association, prospective evidence, especially in FEP populations, is lacking. This hinders early identification of individuals at risk, limits preventive strategies, and constrains informed, personalised treatment decisions.

To address these knowledge gaps, this study aimed to estimate the incidence of PBG and identify its predictors in a prospective cohort of individuals with FEP, particularly the role of third-generation antipsychotics, specifically aripiprazole.

## Methods

Reporting of this study conforms to the Strengthening the Reporting of Observational Studies in Epidemiology statement [22]. The study was registered on ClinicalTrials.gov (NCT05686772), and the protocol has been published [23].

### Study design and setting

This prospective multicentre cohort study was conducted at two EIS for FEP in Quebec, Canada. Both programmes are funded through Quebec’s universal public healthcare system and admit all incident cases of FEP within their designated catchment areas. The Clinique Notre-Dame des Victoires primarily serves Quebec City, the second-largest city in the province of Quebec, as well as surrounding rural areas. The Clinique JAP covers an area located in downtown Montreal, the largest city in the province of Quebec and the second-largest in Canada. Eligibility for admission to either EIS includes: (i) age between 18 and 35 years; (ii) a Diagnostic and Statistical Manual of Mental Disorders, Fifth Edition (DSM-5) diagnosis of FEP, including schizophrenia spectrum disorders or mood disorders with psychotic features; and (iii) antipsychotic treatment exposure of 1 year or less before admission. Individuals with psychosis due to a medical condition or with a severe intellectual disability are ineligible. At the time of the study, the two EIS collectively admitted ~200 new patients annually. Both EIS deliver multidisciplinary care for up to 3 years, in accordance with provincial guidelines for early psychosis intervention [24, 25], centred around a case management model and largely aligned with standards from the National Institute for Health and Care Excellence [26]. Both clinics also benefit from well-integrated research teams.

For this study, patients admitted to these two EIS programmes between November 1, 2019, and January 31, 2023, were followed until the first occurrence of the primary outcome, loss to follow-up, or May 1, 2023. A standardised screening procedure for PBG was implemented across both sites. Assessments were systematically conducted at admission and every 6 months thereafter, or earlier if PBG was suspected. These screenings were carried out by clinical case managers (e.g., nurses, social workers, and occupational therapists) in collaboration with treating psychiatrists. The research teams were not involved in the assessment itself but provided logistical support to ensure its completion (e.g., reminders to clinical teams).

### Study cohort

All patients admitted to the two EIS programmes during the study period were considered for inclusion, which implied meeting the eligibility/ineligibility criteria described above. Exclusion criteria were: (i) active or prior PBG at the time of admission, and (ii) loss to follow-up within the first 3 months.

### Primary outcome

The primary outcome was incident PBG, defined as either a DSM-5 diagnosis of gambling disorder made by the treating psychiatrist or a Problem Gambling Severity Index (PGSI) score ≥ 8. The PGSI is a validated 9-item measure assessing gambling severity, with total scores ranging from 0 to 27: 0 = non-problematic gambling; 1–4 = low-risk; 5–7 = moderate-risk; and ≥ 8 = PBG [27, 28]. In general population samples, PGSI-defined PBG has demonstrated strong convergent validity with DSM-5-defined gambling disorder, with correlation coefficients ranging from 0.89 to 0.96 and a stratum-specific likelihood ratio of 67.9 (95% confidence interval [CI] = 35.6–129.5) [29, 30]. The PGSI has also been the most widely used instrument in studies assessing gambling among individuals with psychotic disorders [13].

Outcome assessment followed a structured three-step process: (i) initial screening for gambling behaviour in the past 6 months (or 12 months at admission), including types of activities and total expenditures; (ii) PGSI administration for individuals who reported gambling and expenditures ≥ CAD 50 in the previous 6 months (or ≥ CAD 100 in the previous 12 months at admission); (iii) DSM-5 diagnostic evaluation for individuals with PGSI scores ≥5. The first two steps were conducted by the patients’ case manager or treating psychiatrist, and the DSM-5 evaluation was performed by the treating psychiatrist using clinical interviews and collateral information from family members. As noted above, assessments could be conducted earlier if the clinical team suspected substantial changes in gambling behaviour, new-onset financial difficulties, or concerns raised by family (as part of the EIS treatment offering) or other sources. The estimated date of PBG onset was determined through clinician and patient reports. Relevant documentation was recorded in medical records, and individuals identified with PBG were offered appropriate clinical support and/or referral to specialised services.

### Covariates

Covariates were selected based on prior literature identifying established or potential associations with PBG [12, 16–18]. Baseline variables included gender identity, self-reported ethnicity, highest level of educational attainment, and prior antipsychotic exposure. Additional variables were collected at baseline and at each 6-month follow-up, including employment, student status, homelessness, psychiatric hospitalisations, primary and comorbid DSM-5 diagnoses, Clinical Global Impressions – Severity score (rated by the treating psychiatrist or a consensus between two trained research assistants) [31], Social and Occupational Functioning Assessment Scale score (rated by the treating psychiatrist or a consensus between two trained research assistants) [32], tobacco smoking, and concomitant psychotropic medication use (antidepressants, benzodiazepines/hypnotics, mood stabilisers, and attention-deficit/hyperactivity disorder [ADHD] medications).

Antipsychotic exposure was recorded on a continuous basis and categorized as current use of: (i) first-generation antipsychotics; (ii) second-generation antipsychotics; (iii) third-generation antipsychotics (dopamine D2/D3–5-HT1A partial agonists); or (iv) a combination thereof. Exposure to third-generation antipsychotics was further disaggregated by specific agent (aripiprazole, brexpiprazole, or cariprazine). Use of adjunctive medications “taken on an as-needed basis” was not recorded.

### Data collection

Data were prospectively extracted from patients’ medical records by the research teams at both study sites using a standardized template. Information was collected at admission and updated every 6 months throughout follow-up. Gambling-related information documented by clinical case managers and treating psychiatrists was also extracted from the medical records by the research teams. All data were entered and managed using the Research Electronic Data Capture platform, hosted locally at each site [33, 34]. As planned a priori, datasets from both sites were merged upon study completion for pooled analysis.

### Statistical analyses

Baseline characteristics were summarised using descriptive statistics. The incidence rate was calculated by dividing the number of incident PBG cases by the total person-time at risk during the follow-up period. Comparisons between individuals with and without incident PBG were conducted using Student’s *t*-test for continuous variables and chi-squared or Fisher’s exact tests for categorical variables, as appropriate. Each potential predictor of PBG was examined in a separate between-individual, time-varying Cox regression model and adjusted for study site as a fixed effect to account for site-level differences and potential confounding (Supplementary Table 1) [35]. Antipsychotic exposure was modelled as a continuously updated time-varying variable, as described above. Follow-up was right-censored at the date of loss to follow-up or end of the observation period. To assess the association between third-generation antipsychotics (aripiprazole, brexpiprazole, and cariprazine), aripiprazole specifically, and PBG, additional models were adjusted for a priori confounders identified using directed acyclic graphs and the back-door criterion, informed by prior literature, as well as clinical and methodological expertise (Supplementary Figures 1 and 2) [36].

Sensitivity analyses were conducted using DSM-5 gambling disorder as an alternative to the composite PBG outcome. Missing data were minimal (<5%) for all covariates included in the multivariable Cox models (Supplementary Table 2). Current tobacco smoking had 22% missing data but was not included in the multivariable models, given its presumed non-confounding role in the association between antipsychotic exposure and PBG. However, missingness in smoking status was associated with the outcome (*p* = .048), suggesting potential informative missingness. No imputation was performed, and complete case analysis was applied. All hazard ratios (HRs) are reported with 95% CIs, and statistical significance was set at *p* < .05. Analyses were performed using R software, version 4.3.0 [37].

### Ethics

Ethical approval was obtained from the institutional review boards at both participating sites (#MP-13-2020-1843). Given the minimal risk involved, a waiver of informed consent was granted, and access to patients’ medical records was authorised by the Commission d’accès à l’information du Québec [Commission for Access to Information of Quebec]. To protect confidentiality, all data were de-identified, and only anonymised datasets were shared between study sites. This study was conducted in accordance with the Declaration of Helsinki.

## Results

### Cohort characteristics and PBG incidence

Of 576 eligible individuals with FEP, 7 (1.2%) were excluded due to a prior history of PBG at admission, and 49 (8.5%) were lost to follow-up within the first 3 months, resulting in a final cohort of 520 patients (mean age 24.6 years; standard deviation [SD] = 4.0; 28.8% women). Over a mean follow-up of 478 days (SD = 282), corresponding to 681.1 person-years of observation, 18 individuals developed incident PBG, including 13 who met DSM-5 criteria for gambling disorder. This represented an incidence rate of 2.6 cases per 100 person-years (95% CI = 1.6–4.2), with no statistically significant difference between study sites (*p* = .566). Among the 18 incident cases, 5 experienced a delay exceeding 3 months between PBG onset and clinical identification despite systematic screening (range 140–457 days). In two of these cases, detection was prompted by information provided by family members or relatives. When including individuals with preexisting PBG at baseline, the overall prevalence was 4.7% (25/527).

At cohort entry, individuals who later developed PBG were more likely to self-identify as of White ethnicity (94.4% vs. 62.6%; *p* = .006), to have not completed high school (55.6% vs. 28.3%; *p* = .013), to have a comorbid ADHD diagnosis (50.0% vs. 25.0%; *p* = .026), and to report non-problematic gambling in the 12 months before entry into EIS (33.3% vs. 13.3%; *p* = .029; Table 1). Only two individuals with incident PBG were identified as women (one cisgender and one transgender). Overall, 84.6% of the cohort had a baseline diagnosis of non-affective psychotic disorder. Most patients (85.4%) had at least one comorbid psychiatric condition, with SUDs being the most common (56.2%). The most frequently observed SUDs were cannabis use disorder (50.6%) and stimulant use disorder (21.1%). Other prevalent comorbidities included personality disorders (29.4%) and ADHD (25.8%). At the time of admission to EIS, 77.3% of patients were receiving antipsychotic treatment.

**Table 1.** Baseline characteristics of the study cohort according to occurrence of problem gambling

	PBG during follow-up			*p-*value
	Yes (*n* = 18) *n* (%)		No (*n* = 502) *n* (%)	
Age, mean ± SD, years	23.2 ± 3.6		24.7 ± 4.0	.126
Female gender	2 (11.1)		146 (29.4)	.092
White ethnicity	17 (94.4)		305 (62.6)	.006
High school not completed	10 (55.6)		133 (28.3)	.013
Occupational status				
Employed	4 (22.2)		164 (33.1)	.333
Student	2 (11.1)		122 (24.6)	.265
In a relationship	5 (27.8)		89 (18.1)	.348
Homelessness	1 (5.6)		61 (12.3)	.711
History of psychiatric hospitalisation	11 (61.1)		312 (63.5)	.833
Main psychiatric diagnosis				
Schizophrenia spectrum psychotic disorder	17 (94.4)		385 (84.2)	.332
Psychotic mood disorder	1 (5.6)		72 (15.8)	
CGI-S score, mean ± SD	3.8 ± 1.7		4.2 ± 1.5	.304
SOFAS score, mean ± SD	49.0 ± 16.0		51.0 ± 13.7	.602
Any comorbid psychiatric diagnosis	16 (94.1)		388 (85.1)	.488
Any substance use disorder	14 (77.8)		278 (55.4)	.060
Alcohol use disorder	3 (16.7)		67 (13.5)	.723
Cannabis use disorder	13 (72.2)		248 (49.8)	.062
Stimulant use disorder	6 (33.3)		103 (20.7)	.236
ADHD	9 (50.0)		125 (25.0)	.026
Anxiety disorder	3 (16.7)		84 (16.7)	1.000
Major depressive disorder	0 (0.0)		25 (5.0)	1.000
Any personality disorder	5 (27.8)		148 (29.5)	.876
Cluster B personality disorder	4 (22.2)		88 (17.6)	.540
Tobacco smoking	10 (55.6)		183 (45.3)	.393
Past exposure to antipsychotic(s)	7 (38.9)		182 (36.3)	.819
Currently using antipsychotic(s)	15 (83.3)		387 (77.1)	.775
Second-generation antipsychotic	12 (66.7)		265 (52.8)	.337
Third-generation antipsychotic	5 (27.8)		158 (31.5)	1.000
Concomitant psychotropic medication				
ADHD medication	2 (11.1)		17 (3.4)	.137
Antidepressant	1 (5.6)		82 (16.3)	.332
Benzodiazepine/hypnotic	2 (11.1)		33 (6.6)	.345
Mood stabiliser	2 (11.1)		30 (6.0)	.305
Gambling in the past 12 months	6 (33.3)		67 (13.3)	.029

Abbreviations: ADHD, attention-deficit hyperactivity disorder; CGI-S, Clinical Global Impressions – Severity scale; PBG, problem gambling; SD, standard deviation; SOFAS, Social and Occupational Functioning Assessment Scale.

### Gambling behaviours

Among the 520 patients, 123 individuals (23.7%) reported gambling in the 12 months before cohort entry and/or at some point during follow-up. Among these gamblers, those who developed PBG were statistically more likely to report online gambling, use of electronic gambling machines (i.e., slot machines and video lottery terminals), card games, and participation in two or more gambling activities (Table 2). Conversely, they were less likely to have gambled in the year preceding admission or to have purchased lotteries. Among those who developed PBG, peak monthly gambling expenditures exceeded CAD 300.

**Table 2.** Gambling activities among all gamblers of the study cohort (*N* = 123)^a^

	PBG during follow-up		*p-*value
	Yes (*n* = 18) *n* (%)	No (*n* = 105) *n* (%)	
Gambling at admission and/or the previous 12 months	6 (33.3)	67 (63.8)	.015
Online gambling	8 (44.4)	18 (17.1)	.024
Lottery	7 (38.9)	71 (67.6)	.019
Electronic gambling machines	11 (61.1)	26 (24.8)	.002
Bingo	0 (0.0)	7 (6.7)	.592
Card games	8 (44.4)	21 (20.0)	.035
Betting	3 (16.7)	18 (17.1)	1.000
Other (e.g., loot boxes)	1 (5.6)	3 (2.9)	.473
Number of gambling formats			
≥ 2	10 (58.8)	30 (30.1)	.021
Maximum amount of money spent on gambling, mean ± SD, *$CAD/month*	309.5 ± 813.9	41.1 ± 124.6	.181
Median (range), *$CAD/month*	83 (13–3500)	8 (1–1000)	

Abbreviations: PBG, problem gambling; SD, standard deviation.

a Individuals who reported gambling at admission and/or at some point during follow-up.

### Predictors of PBG

In the full cohort of 520 patients, statistically significant predictors of incident PBG after adjustment for study site included White ethnicity (HR = 9.73; 95% CI = 1.26–74.8; *p* = .029), incomplete high school education at cohort entry (HR = 2.79; 95% CI = 1.07–7.23; *p* = .035), current stimulant use disorder (HR = 2.76; 95% CI = 1.04–7.30; *p* = .041), current use of third-generation antipsychotics (HR = 4.59; 95% CI = 1.49–14.1; *p* = .008), and a history of non-problematic gambling in the 12 months preceding cohort entry (HR = 3.09; 95% = CI 1.14–8.38; *p* = .026; Table 3). For all other variables examined, the 95% CIs encompassed 1.

**Table 3.** Predictors of problem gambling in the study cohort

	HR^a^	95% CI	*p-*value
Age at baseline	0.89	0.77–1.02	.097
Female gender	0.31	0.07–1.38	.125
White ethnicity	9.73	1.26–74.8	.029
High school not completed at baseline	2.79	1.07–7.23	.035
Employed	0.71	0.26–1.92	.498
Student	0.20	0.03–1.50	.116
In a relationship	1.20	0.39–3.70	.749
Homelessness	2.44	0.51–11.6	.261
Any substance use disorder	2.07	0.74–6.06	.162
Alcohol use disorder	0.85	0.19–3.74	.831
Cannabis use disorder	2.19	0.80–5.98	.125
Stimulant use disorder	2.76	1.04–7.30	.041
ADHD	2.41	0.90–6.43	.080
Anxiety disorder	0.83	0.24–2.90	.770
Major depressive disorder	1.62	0.21–12.3	.641
Cluster B personality disorder	1.28	0.42–3.93	.668
Tobacco smoking	2.25	0.82–6.16	.115
Current use of second-generation antipsychotic	0.86	0.33–2.25	.765
Current use of third-generation antipsychotic	4.59	1.49–14.1	.008
Gambling at admission and/or the previous 12 months	3.09	1.14–8.38	.026

Abbreviations: ADHD, attention-deficit hyperactivity disorder; CI, confidence interval; HR, hazard ratio.

a Hazard ratios are adjusted for site only.

Among the 18 cases of incident PBG, 13 occurred during treatment with aripiprazole, 9 as monotherapy, and 4 in combination with a second-generation antipsychotic. Of these 13 cases, 4 patients were receiving the oral formulation of aripiprazole and 9 patients were receiving the long-acting injectable formulation, with a mean oral-equivalent dose of 11.3 mg/day (SD = 3.8) at the time of PBG onset. Among the five remaining cases, three occurred during treatment with second-generation antipsychotics, one during combined treatment with the third-generation antipsychotic cariprazine and a second-generation antipsychotic, and one in the absence of antipsychotic treatment. In fully adjusted models accounting for study site, age, gender, ethnicity, anxiety disorder, cluster B personality disorders, major depressive disorder, SUDs, and prior non-problematic gambling, current use of third-generation antipsychotics remained significantly associated with PBG (adjusted HR [aHR] = 6.06; 95% CI = 1.90–19.4; *p* = .002; Table 4). A statistically significant association was also observed for aripiprazole specifically (aHR = 4.66; 95% CI = 1.57–13.9; *p* = .006). For first- or second-generation antipsychotics, the 95% CI of the aHR included 1.

**Table 4.** Risk of problem gambling with the use of different antipsychotics

	Users	Events	Person-years	Adjusted HR^a^	95% CI	*p-*value
Current use of first-/second-generation antipsychotics	362	8	354.0	0.69	0.26–1.84	.462
Current use of third-generation antipsychotics	296	14	276.7	6.06	1.90–19.4	.002
Current use of aripiprazole	289	13	268.7	4.66	1.57–13.9	.006

Abbreviations: CI, confidence interval; HR, hazard ratio.

a Hazard ratios are adjusted for age (baseline), gender (baseline), ethnicity (baseline), anxiety disorder, cluster B personality disorder, major depressive disorder, substance use disorder, gambling at admission and/or the previous 12 months, and site.

### Sensitivity analyses

As previously described, sensitivity analyses were conducted using DSM-5 gambling disorder in place of the composite PBG outcome. Among the 13 individuals with incident DSM-5 gambling disorder, the only baseline variable that differed significantly from the rest of the cohort was self-reported White ethnicity (92.3% vs. 63.0%; *p* = .038; Supplementary Table 3). In models adjusted for study site, current use of third-generation antipsychotics was associated with an increased risk of gambling disorder (HR = 15.6; 95% CI = 2.00–120.8; *p* = .009; Supplementary Table 4). For all other factors for which HRs could be computed, the 95% CIs encompassed 1.

Of the 13 gambling disorder cases, 12 occurred during treatment with aripiprazole, 8 as monotherapy, and 4 with concurrent use of a second-generation antipsychotic. The remaining case occurred during treatment with a second-generation antipsychotic. In fully adjusted models, current use of third-generation antipsychotics (aHR = 19.6; 95% CI = 2.44–156.5; *p* = .005) and aripiprazole specifically (aHR = 20.3; 95% CI = 2.54–162.5; *p* = .005) were associated with increased risks of gambling disorder (Supplementary Table 5). No statistically significant association was observed for first- or second-generation antipsychotics.

## Discussion

### Summary of main findings

This is the first prospective cohort study to examine the incidence and predictors of PBG in a sample representative of the majority of individuals with FEP within defined catchment areas. Among 520 young adults with FEP followed for an average of 1.3 years, the incidence of PBG was 2.6 cases per 100 person-years. Independent predictors of incident PBG included White ethnicity, incomplete high school education, current stimulant use disorder, non-problematic gambling prior to admission, and exposure to third-generation antipsychotics (dopamine D2/D3–5-HT1A partial agonists). After adjustment for relevant confounders, aripiprazole was associated with a 4.7-fold (95% CI = 1.6–13.9) increased risk of PBG.

### Comparison with previous literature

The observed lifetime PBG prevalence of 4.7% was lower than the 8.7% pooled estimate from a recent meta-analysis of cross-sectional and retrospective studies among individuals with psychotic disorders, but consistent with methodologically rigorous studies using validated screening instruments such as the PGSI [13]. For instance, a previous nested case–control study conducted at the Quebec study site reported a PBG prevalence of 6.4% among individuals with FEP [18]. In contrast, the general population prevalence of PBG in Canada has been estimated at 0.3% in 2018 [38], suggesting a substantially elevated risk in FEP.

#### Educational attainment

Similar to the general population where lower educational attainment has been linked to a greater risk of PBG [17], in our study, individuals who had not completed high school had an increased risk of developing PBG. This finding is consistent with limited prior evidence suggesting that, among individuals with psychotic disorders, those with moderate-risk or PBG were twice as likely to have not reached a high school diploma compared to non-problematic gamblers [39]. Lower educational attainment may reflect broader psychosocial adversity, including reduced health literacy, limited employment opportunities, and heightened vulnerability to addiction. Although ADHD was not independently associated with PBG after adjustment, it was more prevalent among individuals who developed PBG. Given its associations with academic underachievement, executive dysfunction, and impulsivity, ADHD could also serve as a mediating factor linking low educational attainment to increased gambling risk [40]. This hypothesis aligns with findings from general population studies [41, 42] and warrants further exploration in psychosis-specific cohorts.

#### Stimulant use disorder and impulsivity

Stimulant use disorder also emerged as an independent predictor of PBG, supporting the conceptual and neurobiological overlap between behavioural and substance-related addictions. Although this might simply reflect broader psychosocial adversity consequences, limited evidence specific to individuals with psychotic disorders has shown higher rates of substance use and SUDs, including alcohol, cannabis, tobacco, and stimulants, among those with moderate-risk or PBG in this population [39, 43–45]. Shared pathophysiological mechanisms, such as reward system dysregulation and heightened impulsivity, may underlie these associations [16, 46]. Although impulsivity was not directly assessed in our study, its well-established role in both PBG and SUDs suggests it may have contributed to the observed relationship [17, 47]. In this context, comorbid ADHD and SUDs may possibly serve as indirect indicators of trait impulsivity, although this should be further studied. While cluster B personality disorders, frequently linked to impulsivity, were not independently associated with PBG in our cohort, they have been commonly reported in case series of aripiprazole-associated impulse control disorders and PBG [20, 48].

#### Antipsychotic treatment and PBG risk

Exposure to third-generation antipsychotics, particularly aripiprazole, was associated with a significantly increased risk of incident PBG. This is consistent with prior pharmacovigilance studies and case series linking aripiprazole and other partial dopamine agonists to impulse control disorders, including hypersexuality and compulsive shopping, and PBG [20, 48, 49]. Our previous nested case–control study in FEP patients reported a similar association with aripiprazole, though it was limited by its retrospective design and small sample size [18]. Third-generation antipsychotics share partial agonist activity at dopamine D3 receptors, implicated in reward processing and motivational salience, as well as serotonin 5-HT1A receptors, involved in impulse control via the dorsal raphe nucleus. A comparable phenomenon has been documented in Parkinson’s disease, where dopamine agonists with high affinity for D3 receptors have been linked to the onset of PBG and impulse control disorders [50–52]. Although this mechanism provides biological plausibility for our findings, a pharmacovigilance-pharmacodynamic analysis did not find an association between antipsychotics’ D3 affinity and impulse control disorders; instead, the signal pointed toward 5-HT1A receptor agonism, a result that requires further replication [53]. Taken together, these observations suggest that overlapping dopaminergic and serotonergic mechanisms may contribute to the emergence of PBG in susceptible individuals treated with partial dopamine agonists. While people prescribed third-generation antipsychotics may display traits such as impulsivity or comorbidities such as SUDs that increase vulnerability to PBG, our adjusted models incorporated several related clinical correlates (including anxiety disorders, depression, cluster B personality disorders, SUDs, and prior non-problematic gambling), and the association persisted, suggesting that differential prescribing on this basis is unlikely to fully account for the findings.

However, the increased risk of PBG identified in this study must be carefully balanced against the clinically important benefits of aripiprazole in FEP populations. Aripiprazole is one of the few non-first-generation antipsychotics available in a long-acting injectable formulation, which is associated with improved treatment adherence and a reduced risk of relapse in both chronic schizophrenia [6] and FEP [54]. It is also characterised by a favourable tolerability profile, including a lower cardiometabolic burden, prolactin-sparing effects, and a reduced incidence of movement disorders compared to many other antipsychotics [55]. As such, aripiprazole remains an important therapeutic option in early psychosis, often considered a first-line pharmacological treatment.

#### Sociodemographic factors

White ethnicity was associated with an increased risk of PBG in this study. This finding diverges from some previous studies suggesting higher gambling rates among ethnic minority groups [16, 56], though such associations may be context-dependent and shaped by intersecting factors such as socioeconomic status and cultural attitudes toward gambling [57]. Further research is needed to disentangle these complex relationships, specifically among individuals with psychotic disorders. Although the overall prevalence of PBG was numerically higher in men than in women, no significant associations were found between PBG risk and either gender or age in our cohort. This contrasts with general population evidence, where male sex and younger age are consistently associated with increased PBG risk, although in the case of gender, most prior studies have focused on biological sex rather than gender identity [16, 17, 58, 59]. The absence of significant gender or age effects in our study likely reflects the limited variability of these characteristics within FEP populations, which are predominantly composed of young men, thereby reducing the statistical power to detect more subtle associations.

#### Prior gambling and gambling modalities

A history of non-problematic gambling in the 12 months preceding admission predicted incident PBG, potentially reflecting a multifactorial vulnerability that includes greater familiarity with gambling, increased exposure to risk environments, or the presence of under-recognised problematic patterns before entry into EIS. Interestingly, among individuals who reported any gambling during the study period, those who developed PBG were less likely to have reported gambling at cohort entry, suggesting that gambling may have been initiated for the first time during follow-up in some cases. This finding raises the possibility that emerging clinical vulnerabilities or treatment-related factors, such as medication effects or evolving psychosocial stressors, may have contributed to the onset of gambling behaviour. Alternatively, the age range of the sample may also explain a greater probability of exposure to gambling during follow-up, as gambling is prohibited before the age of 18 years in the province of Quebec.

Exploratory results also indicated that individuals who developed PBG were more likely to engage in gambling modalities with elevated addictive potential, including online gambling, card games, and electronic gambling machines, whereas lottery play was more common among those who did not develop PBG. These patterns mirror findings from general population studies and may reflect the influence of structural characteristics such as rapid event frequency and continuous reinforcement schedules [16, 60]. Further research is needed to understand gambling motivations in individuals with psychotic disorders, particularly in the early stages of FEP, and to identify the factors that facilitate the transition from recreational to problematic gambling in this high-risk population.

### Potential mechanisms linking psychotic disorders and PBG

Several complementary mechanisms may contribute to an elevated risk of PBG in individuals with psychotic disorders. Longitudinal evidence indicates that psychotic disorders typically precede the onset of PBG, suggesting that gambling problems tend to arise against a background of preexisting neurobiological and psychosocial vulnerabilities associated with psychosis, rather than serving as a primary trigger of psychosis [18, 61–63]. Individuals with psychotic disorders are disproportionately exposed to childhood adversity, substance use, socioeconomic disadvantage, and low educational attainment, factors that are also associated with increased PBG risk [16, 17, 47, 64, 65]. Genetic vulnerability may further contribute to this association, as emerging evidence suggests that polygenic liability for schizophrenia is modestly associated with PBG [66]. However, this relationship may be indirect, as the polygenic risk for schizophrenia is also associated with increased susceptibility to substance use [67–69], which may in turn mediate part of the heightened risk of PBG [16, 70].

At the neurobiological level, psychotic disorders are characterised by dysregulation of mesolimbic dopamine signalling, including ventral striatal hypoactivation during reward anticipation, reflecting impaired salience processing [71–73], alongside hypoactivity in prefrontal regions involved in cognitive control and decision-making [74]. These alterations may reduce the ability to discriminate salient from neutral cues and to regulate reward-seeking behaviour [75]. In contrast, PBG has been associated with hyperactivation of ventral and dorsal striatal regions and altered prefrontal engagement during gambling tasks, reflecting heightened reward sensitivity and impaired top-down control [76, 77]. Gambling-related cues may therefore act as particularly potent stimuli capable of overriding already compromised salience attribution and inhibitory control in individuals with psychotic disorders. Together, these convergent disturbances in reward processing, learning, and inhibitory control may create a neurobiological vulnerability pathway through which individuals with psychotic disorders are particularly susceptible to developing PBG. Beyond dopaminergic dysfunction, serotonergic and glutamatergic abnormalities, particularly N-methyl-D-aspartate (NMDA) receptor hypofunction, have also been implicated in both psychosis and addictive disorders, and may represent additional shared mechanisms contributing to this comorbidity [78–80].

### Strengths and limitations

This study has several methodological strengths. Its prospective design enabled the temporal sequencing of exposures and outcomes, thereby reducing the risk of recall bias and enhancing causal inference. Systematic PBG screening and assessment were embedded into routine clinical care at two distinct EIS, strengthening ecological validity and generalisability to real-world mental health settings. The use of both the PGSI and DSM-5 diagnostic criteria ensured comprehensive and validated outcome measurement, while diagnoses were made by treating psychiatrists with access to rich clinical information and collateral reports, including families, enhancing diagnostic accuracy. Another strength lies in the integration of detailed longitudinal clinical, sociodemographic, and pharmacologic data, which allowed for nuanced modelling of time-varying exposures such as antipsychotic use. Importantly, confounders were selected based on the directed acyclic graphs approach [36], informed by clinical expertise and prior literature, enhancing the credibility of causal inference.

Nevertheless, some limitations must also be acknowledged. First, the relatively small number of PBG cases observed reduced the statistical power to detect certain associations and contributed to wide 95% CIs for some estimates. This limited power may also partly explain discrepancies between our findings and those reported in general population samples. Second, impulsivity, a well-established risk factor for PBG, was not directly measured. As a result, full confounder control was not possible, and some degree of residual confounding cannot be excluded. Accordingly, as this was an observational study, the reported associations should be interpreted as correlational and do not establish causality. Third, although the primary outcome combined DSM-5-defined gambling disorder and PGSI-defined PBG, as both measures are validated and associated with meaningful functional and psychosocial consequences, it is not impossible that this composite outcome may have captured individuals with differing clinical profiles. However, sensitivity analyses restricted to DSM-5-defined cases produced consistent results regarding the associations with third-generation antipsychotics and aripiprazole specifically, supporting the validity and robustness of the main findings. Fourth, although missing data were minimal for covariates included in the multivariable models, current tobacco smoking had a higher rate of missingness (22%), and this missingness was associated with the outcome. While smoking was not included in the adjusted models, because it was not identified as a confounder of the association between antipsychotic exposure and PBG based on the DAG approach, it limited the ability to evaluate smoking as an independent predictor or potential effect modifier. The possibility of informative missingness therefore remains, and future studies should ensure more comprehensive capture of smoking status to explore its potential role in the development of PBG in early psychosis.

Finally, delays of several months between the estimated onset of PBG and its clinical detection were observed in some cases, despite systematic screening procedures. This suggests that the incidence estimates may be conservative and highlights the likelihood of under-recognition or underreporting of emerging gambling-related behaviours in routine care. Such delays may also have limited the precision of onset dating, emphasising the need for more sensitive or continuous monitoring approaches in future studies. Several individuals appeared to underreport or fail to recognise the problematic nature of their gambling behaviour, even when directly questioned. This underreporting may reflect factors such as shame, fear of judgement, a desire to manage the problem without help, or internalised stigma related to gambling and mental illness more broadly [81]. These findings underscore the importance of systematic, longitudinal PBG screening within EIS for FEP. When possible, collateral information from family members or close contacts may enhance early recognition and improve assessment accuracy.

### Implications

Although potentially underestimated, PBG was not uncommon in this prospective, real-world cohort of individuals with FEP and was associated with modifiable risk factors, including stimulant use disorder and, most notably, treatment with third-generation antipsychotics, particularly aripiprazole. In line with the principle of “primum non nocere,” systematic screening for PBG should be integrated into early psychosis care, especially when prescribing aripiprazole or other third-generation antipsychotics. Treatment planning should carefully weigh the clinical benefits of these medications against potential risks, considering individual vulnerability profiles to ensure safer, more personalised care. Early identification and intervention for this often iatrogenically triggered comorbidity may help to prevent long-term functional impairment and support recovery.

## Supporting information

10.1192/j.eurpsy.2026.10167.sm001 Corbeil et al. supplementary material Corbeil et al. supplementary material

## Data Availability

Due to the absence of participant consent for public data sharing, the datasets generated and analysed during the current study are not publicly available. Researchers interested in the study methods or aggregated findings may contact the corresponding author for further information.

## Supplementary material

The supplementary material for this article can be found at http://doi.org/10.1192/j.eurpsy.2026.10167.
