# Supplementary material for: Incidence and predictors of problem gambling in first-episode psychosis: A prospective multicentre cohort study
Source: Eur Psychiatry. 2026 Feb 13;69(1):e29. doi: 10.1192/j.eurpsy.2026.10167 (PMC13122518; doi:10.1192/j.eurpsy.2026.10167)
Supplement: Corbeil et al. supplementary material [file S0924933826101679sup001.docx]

Supplementary material

**Incidence and predictors of problem gambling in first-episode psychosis: a prospective multicenter cohort study**

Olivier Corbeil^1,2,3^, Maxime Huot-Lavoie^3,4^, Amal Abdel-Baki^5,6,7^, Laurent Béchard^1,2,3,8^, Sébastien Brodeur^2,3,4^, Laurence Artaud^5,6,7^, Prométhéas Constantinides^5,6,7^, Christian Jacques^9,10^, Jean-François Morin^5,6,7^, Clairélaine Ouellet-Plamondon^5,6,7^, Marco Solmi^11-14^, Denis Talbot^15,16^, Michel Dorval^1,17^, Isabelle Giroux^9,10^, Marc-André Roy^2,3,4^, Marie-France Demers^1,2,3^

1. Faculty of Pharmacy, Université Laval, Quebec, Canada.

2. First-episode psychosis program, Institut universitaire en santé mentale de Québec (IUSMQ), Quebec, Canada.

3. CERVO Brain Research Centre, Quebec, Canada.

4. Department of Psychiatry, Faculty of Medicine, Université Laval, Quebec, Canada.

5. Département de psychiatrie et d’addictologie, Faculty of Medicine, Université de Montréal, Quebec, Canada.

6. Centre de recherche du Centre hospitalier de l’Université de Montréal (CRCHUM), Quebec, Canada.

7. Youth mental health service, Department of Psychiatry, Centre hospitalier de l’université de Montréal (CHUM), Quebec, Canada.

8. Faculty of Nursing, Université Laval, Quebec, Canada.

9. École de psychologie, Université Laval, Quebec, Canada.

10. Centre québécois d’excellence pour la prévention et le traitement du jeu, Quebec, Canada.

11. SCIENCES lab, Department of Psychiatry, University of Ottawa, Ontario, Canada.

12. Regional Centre for the Treatment of Eating Disorders and On Track: The Champlain First Episode Psychosis Program, Department of Mental Health, The Ottawa Hospital, Ontario, Canada.

13. Ottawa Hospital Research Institute (OHRI), Clinical Epidemiology Program, University of Ottawa, Ontario, Canada.

14. Department of Child and Adolescent Psychiatry, Charité Universitätsmedizin, Berlin, Germany.

15. Department of Social and Preventive Medicine, Faculty of Medicine, Université Laval, Quebec, Canada.

16. CHU de Québec – Université Laval Research Centre, Population Health and Optimal Health Practices Program, Quebec, Canada.

17. CHU de Québec – Université Laval Research Centre, Oncology Program, Quebec, Canada.

**Corresponding author:** Olivier Corbeil, PharmD, MSc

1050, Avenue de la médecine, Québec (Québec), Canada;

Phone number: 1-418-656-3211, fax number: 1-418-656-2305;

Email: [olivier.corbeil.1@ulaval.ca](mailto:olivier.corbeil.1@ulaval.ca)

**Contents**

Supplementary Figure 1. Directed acyclic graph of the unadjusted causal path between aripiprazole use and problem gambling 3

Supplementary Figure 2. Directed acyclic graph of the adjusted causal path between aripiprazole use and problem gambling 4

Supplementary Table 1. Baseline characteristics of the study cohort according to study site 5

Supplementary Table 2. Proportion of missing values for putative predictors of problem gambling in the study cohort 6

Supplementary Table 3. Baseline characteristics of the study cohort according to occurrence of gambling disorder 7

Supplementary Table 4. Predictors of gambling disorder diagnosis in the study cohort 8

Supplementary Table 5. Risk of gambling disorder diagnosis with the use of different antipsychotics 9


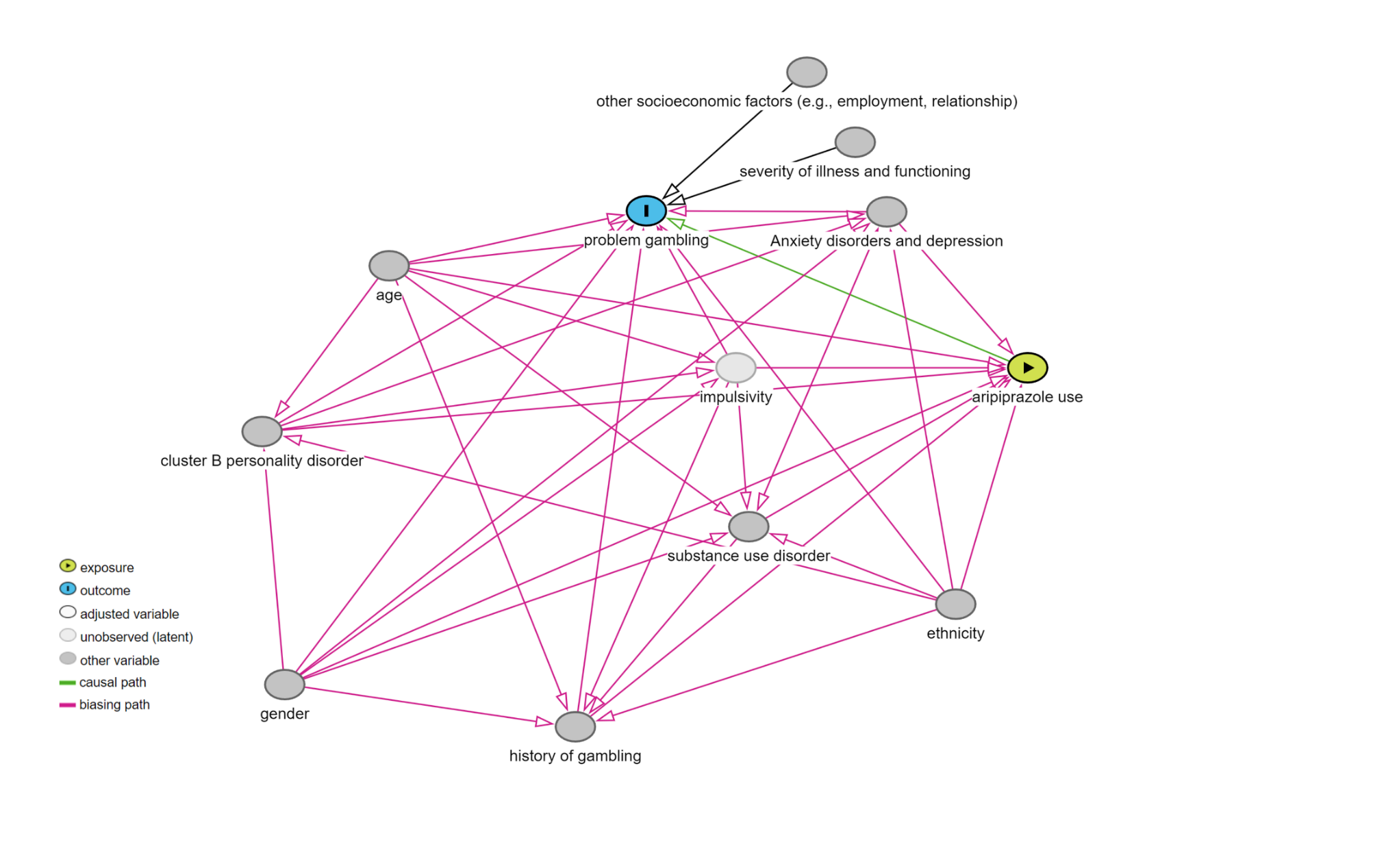


Supplementary Figure 1. Directed acyclic graph of the unadjusted causal path between aripiprazole use and problem gambling

Generated from: Textor J, van der Zander B, Gilthorpe MS, Liskiewicz M, Ellison GT. Robust causal inference using directed acyclic graphs: the R package 'dagitty'. Int J Epidemiol. 2016;45(6):1887-94.


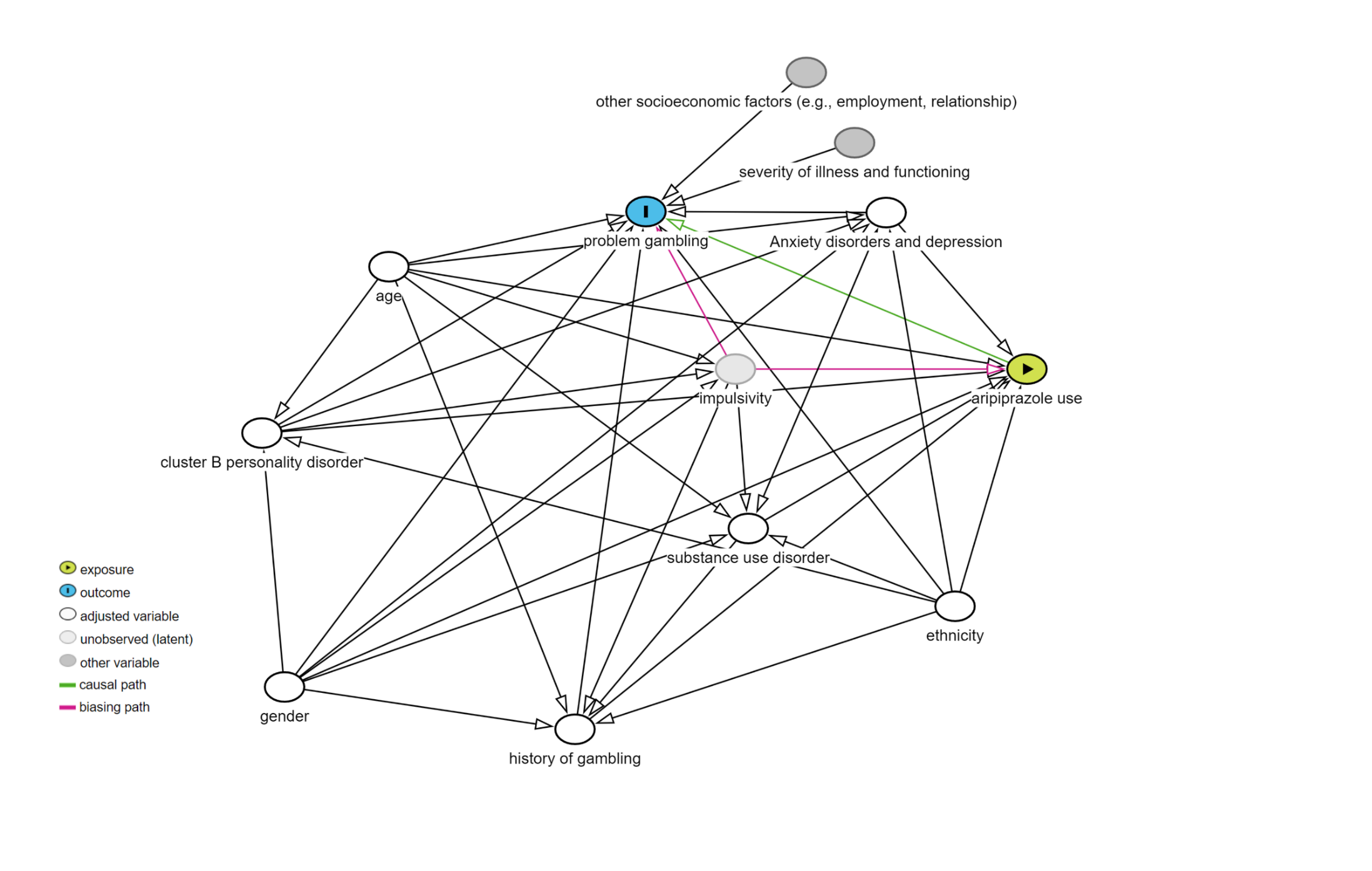


Supplementary Figure 2. Directed acyclic graph of the adjusted causal path between aripiprazole use and problem gambling

Generated from: Textor J, van der Zander B, Gilthorpe MS, Liskiewicz M, Ellison GT. Robust causal inference using directed acyclic graphs: the R package 'dagitty'. Int J Epidemiol. 2016;45(6):1887-94.

Supplementary Table 1. Baseline characteristics of the study cohort according to study site

|  | Study site 1  (n = 292) |  | Study site 2  (n = 228) | *p* value |
| --- | --- | --- | --- | --- |
|  | n (%) |  | n (%) |  |
| Age, mean ± SD, *years* | 24.8 ± 4.3 |  | 24.4 ± 3.6 | .205 |
| Female gender | 80 (27.4) |  | 68 (30.6) | .423 |
| White ethnicity | 216 (75.0) |  | 106 (48.4) | < .001 |
| High school not completed | 83 (31.4) |  | 60 (26.8) | .260 |
| Occupational status |  |  |  |  |
| Employed | 113 (39.2) |  | 55 (24.4) | < .001 |
| Student | 65 (22.5) |  | 59 (26.2) | .327 |
| In a relationship | 56 (19.4) |  | 38 (17.2) | .529 |
| Homelessness | 10 (3.4) |  | 52 (23.3) | < .001 |
| History of psychiatric hospitalisation | 236 (81.1) |  | 87 (39.9) | < .001 |
| Main psychiatric diagnosis |  |  |  |  |
| Schizophrenia spectrum psychotic disorder | 244 (84.7) |  | 158 (84.5) | .946 |
| Psychotic mood disorder | 44 (15.3) |  | 29 (15.5) |  |
| CGI-S score, mean ± SD | 3.4 ± 1.4 |  | 5.2 ± 1.0 | < .001 |
| SOFAS score, mean ± SD | 54.2 ± 14.6 |  | 47.7 ± 12.2 | < .001 |
| Any comorbid psychiatric diagnosis | 241 (82.5) |  | 163 (90.1) | .024 |
| Any substance use disorder | 181 (62.0) |  | 111 (48.7) | .002 |
| Alcohol use disorder | 47 (16.1) |  | 23 (10.3) | .055 |
| Cannabis use disorder | 162 (55.5) |  | 99 (44.2) | .011 |
| Stimulant use disorder | 76 (26.0) |  | 33 (14.7) | .002 |
| ADHD | 101 (34.6) |  | 33 (14.5) | < .001 |
| Anxiety disorder | 56 (19.2) |  | 31 (13.6) | .091 |
| Major depressive disorder | 18 (6.2) |  | 7 (3.1) | .111 |
| Any personality disorder | 76 (26.0) |  | 77 (33.8) | .054 |
| Cluster B personality disorder | 63 (21.6) |  | 29 (12.8) | .009 |
| Tobacco smoking | 120 (42.6) |  | 73 (52.1) | .063 |
| Past exposure to antipsychotic(s) | 154 (52.7) |  | 35 (15.4) | < .001 |
| Currently using antipsychotic(s) | 248 (84.9) |  | 154 (67.5) | < .001 |
| Second-generation antipsychotic | 157 (53.8) |  | 120 (52.6) | .797 |
| Third-generation antipsychotic | 116 (39.7) |  | 47 (20.6) | < .001 |
| Concomitant psychotropic medication |  |  |  |  |
| ADHD medication | 9 (3.1) |  | 10 (4.4) | .432 |
| Antidepressant | 53 (18.2) |  | 30 (13.2) | .123 |
| Benzodiazepine/hypnotic | 32 (11.0) |  | 3 (1.3) | < .001 |
| Mood stabiliser | 16 (5.5) |  | 16 (7.0) | .469 |
| Gambling in the past 12 months | 41 (14.0) |  | 32 (14.0) | .998 |

Abbreviations: ADHD, attention-deficit hyperactivity disorder; CGI-S, Clinical Global Impressions – Severity scale; SD, standard deviation; SOFAS, Social and Occupational Functioning Assessment Scale.

Supplementary Table 2. Proportion of missing values for putative predictors of problem gambling in the study cohort

|  | Missing values  n (%)* |
| --- | --- |
| Age (baseline/current) | 0 (0.0) |
| Gender (current) | 23 (1.1) |
| Ethnicity (baseline) | 50 (2.5) |
| High school not completed (baseline) | 90 (4.5) |
| Employed (current) | 45 (2.2) |
| Student (current) | 41 (2.0) |
| In a relationship (current) | 55 (2.7) |
| Homelessness (current) | 46 (2.3) |
| Any substance use disorder (current) | 1 (0.0) |
| Alcohol use disorder (current) | 9 (0.4) |
| Cannabis use disorder (current) | 9 (0.4) |
| Stimulant use disorder (current) | 9 (0.4) |
| ADHD (current) | 1 (0.0) |
| Anxiety disorder (current) | 0 (0.0) |
| Major depressive disorder (current) | 8 (0.4) |
| Cluster B personality disorder (current) | 1 (0.0) |
| Tobacco smoking (current) | 442 (21.9) |
| Use of second-generation antipsychotic (current) | 0 (0.0) |
| Use of first- or second-generation antipsychotic (current) | 0 (0.0) |
| Use of third-generation antipsychotic (current) | 0 (0.0) |
| Use of aripiprazole (current) | 0 (0.0) |
| Gambling at admission and/or previous 12 months | 0 (0.0) |

*Total number of observation periods = 2014.

Supplementary Table 3. Baseline characteristics of the study cohort according to occurrence of gambling disorder

|  | GD during follow-up | | | *p* value |
| --- | --- | --- | --- | --- |
|  | Yes  (n = 13) |  | No  (n = 507) |  |
|  | n (%) |  | n (%) |  |
| Age, mean ± SD, *years* | 23.0 ± 3.5 |  | 24.7 ± 4.0 | .139 |
| Female gender | 2 (15.4) |  | 146 (29.1) | .365 |
| White ethnicity | 12 (92.3) |  | 310 (63.0) | .038 |
| High school not completed | 7 (53.8) |  | 136 (28.6) | .063 |
| Occupational status |  |  |  |  |
| Employed | 4 (30.8) |  | 164 (32.8) | 1.000 |
| Student | 1 (7.7) |  | 123 (24.6) | .205 |
| In a relationship | 3 (23.1) |  | 91 (18.3) | .715 |
| Homelessness | 1 (7.7) |  | 61 (12.2) | 1.000 |
| History of psychiatric hospitalisation | 7 (53.8) |  | 316 (63.7) | .562 |
| Main psychiatric diagnosis |  |  |  |  |
| Schizophrenia spectrum psychotic disorder | 13 (100.0) |  | 389 (84.2) | .234 |
| Psychotic mood disorder | 0 (0.0) |  | 73 (15.8) |  |
| CGI-S score, mean ± SD | 4.0 ± 1.5 |  | 4.2 ± 1.5 | .635 |
| SOFAS score, mean ± SD | 44.4 ± 8.7 |  | 51.1 ± 13.9 | .132 |
| Any comorbid psychiatric diagnosis | 11 (91.7) |  | 393 (85.2) | 1.000 |
| Any substance use disorder | 9 (69.2) |  | 283 (55.8) | .336 |
| Alcohol use disorder | 2 (15.4) |  | 68 (13.5) | .692 |
| Cannabis use disorder | 9 (69.2) |  | 252 (50.1) | .173 |
| Stimulant use disorder | 4 (30.8) |  | 105 (20.9) | .488 |
| ADHD | 6 (46.2) |  | 128 (25.3) | .109 |
| Anxiety disorder | 3 (23.1) |  | 84 (16.6) | .464 |
| Major depressive disorder | 0 (0.0) |  | 25 (5.0) | 1.000 |
| Any personality disorder | 3 (23.1) |  | 150 (29.6) | .764 |
| Cluster B personality disorder | 2 (15.4) |  | 90 (17.8) | 1.000 |
| Tobacco smoking | 7 (53.8) |  | 186 (45.5) | .551 |
| Past exposure to antipsychotic(s) | 4 (30.8) |  | 185 (36.5) | .777 |
| Currently using antipsychotic(s) | 10 (76.9) |  | 392 (77.3) | 1.000 |
| Second-generation antipsychotic | 7 (53.8) |  | 270 (53.3) | .966 |
| Third-generation antipsychotic | 4 (30.8) |  | 159 (31.4) | 1.000 |
| Concomitant psychotropic medication |  |  |  |  |
| ADHD medication | 1 (7.7) |  | 18 (3.6) | .387 |
| Antidepressant | 1 (7.7) |  | 82 (16.2) | .703 |
| Benzodiazepine/hypnotic | 1 (7.7) |  | 34 (6.7) | .600 |
| Mood stabiliser | 1 (7.7) |  | 31 (6.1) | .566 |
| Gambling in the past 12 months | 3 (23.1) |  | 70 (13.8) | .407 |

Abbreviations: ADHD, attention-deficit hyperactivity disorder; CGI-S, Clinical Global Impressions – Severity scale; GD, gambling disorder; SD, standard deviation; SOFAS, Social and Occupational Functioning Assessment Scale.

Supplementary Table 4. Predictors of gambling disorder diagnosis in the study cohort

|  | HR^a^ | 95% CI | | | *p* value |
| --- | --- | --- | --- | --- | --- |
| Age at baseline | 0.86 | 0.72 | – | 1.02 | .090 |
| Female gender | 0.46 | 0.10 | – | 2.09 | .313 |
| White ethnicity | 7.83 | 0.99 | – | 62.9 | .051 |
| High school not completed at baseline | 2.51 | 0.81 | – | 7.78 | .112 |
| Employed | 1.63 | 0.49 | – | 5.40 | .422 |
| Student | - | - | – | - | - |
| In a relationship | 0.77 | 0.17 | – | 3.51 | .731 |
| Homelessness | 2.84 | 0.56 | – | 14.5 | .209 |
| Any substance use disorder | 1.87 | 0.56 | – | 6.26 | .311 |
| Alcohol use disorder | 0.64 | 0.08 | – | 4.96 | .666 |
| Cannabis use disorder | 2.54 | 0.76 | – | 8.53 | .130 |
| Stimulant use disorder | 2.11 | 0.63 | – | 7.06 | .224 |
| ADHD | 2.21 | 0.67 | – | 7.23 | .191 |
| Anxiety disorder | 1.36 | 0.37 | – | 5.07 | .645 |
| Major depressive disorder | 2.44 | 0.31 | – | 19.0 | .394 |
| Cluster B personality disorder | 0.88 | 0.19 | – | 4.00 | .864 |
| Tobacco smoking | 2.37 | 0.70 | – | 8.01 | .164 |
| Current use of second-generation antipsychotic | 0.65 | 0.20 | – | 2.05 | .458 |
| Current use of third-generation antipsychotic | 15.6 | 2.00 | – | 120.8 | .009 |
| Gambling at admission and/or previous 12 months | 1.81 | 0.49 | – | 6.71 | .373 |

Abbreviations: -, non-computable; ADHD, attention-deficit hyperactivity disorder; CI, confidence interval; HR, hazard ratio.

^a^Hazard ratios are adjusted for site.

Supplementary Table 5. Risk of gambling disorder diagnosis with the use of different antipsychotics

|  | Users | Events | Person-years | Adjusted HR^a^ | 95% CI | *p* value |
| --- | --- | --- | --- | --- | --- | --- |
| Current use of first-/second-generation antipsychotics | 362 | 5 | 354.0 | 0.59 | 0.18 – 1.89 | .372 |
| Current use of third-generation antipsychotics | 296 | 12 | 276.7 | 19.6 | 2.44 – 156.5 | .005 |
| Current use of aripiprazole | 289 | 12 | 268.7 | 20.3 | 2.54 – 162.5 | .005 |

Abbreviations: CI, confidence interval; HR, hazard ratio.

^a^Hazard ratios are adjusted for age (baseline), gender (baseline), ethnicity (baseline), anxiety disorder, cluster B personality disorder, major depressive disorder, substance use disorder, gambling at admission and/or previous 12 months, and site.
